# Binocular responsiveness of projection neurons of the praying mantis optic lobe in the frontal visual field

**DOI:** 10.1007/s00359-020-01405-x

**Published:** 2020-02-22

**Authors:** Ronny Rosner, Ghaith Tarawneh, Veronika Lukyanova, Jenny C. A. Read

**Affiliations:** Biosciences Institute, Henry Wellcome Building for Neuroecology, Newcastle University, Framlington Place, Newcastle upon Tyne, NE2 4HH UK

**Keywords:** Insect stereopsis, Praying mantis, 3D vision, Binocular vision, Depth perception

## Abstract

Praying mantids are the only insects proven to have stereoscopic vision (stereopsis): the ability to perceive depth from the slightly shifted images seen by the two eyes. Recently, the first neurons likely to be involved in mantis stereopsis were described and a speculative neuronal circuit suggested. Here we further investigate classes of neurons in the lobula complex of the praying mantis brain and their tuning to stereoscopically-defined depth. We used sharp electrode recordings with tracer injections to identify visual projection neurons with input in the optic lobe and output in the central brain. In order to measure binocular response fields of the cells the animals watched a vertical bar stimulus in a 3D insect cinema during recordings. We describe the binocular tuning of 19 neurons projecting from the lobula complex and the medulla to central brain areas. The majority of neurons (12/19) were binocular and had receptive fields for both eyes that overlapped in the frontal region. Thus, these neurons could be involved in mantis stereopsis. We also find that neurons preferring different contrast polarity (bright vs dark) tend to be segregated in the mantis lobula complex, reminiscent of the segregation for small targets and widefield motion in mantids and other insects.

**Electronic supplementary material:**

The online version of this article (10.1007/s00359-020-01405-x) contains supplementary material, which is available to authorized users.

## Introduction

Stereoscopic vision is a capability found in a range of vertebrate groups but it has so far only been demonstrated in a single insect, the praying mantis (Pettigrew 1986; Nityananda and Read 2017). Mantids are predatory insects: if prey is in catching range the animals snatch it with their raptorial front legs. They use stereoscopic vision to estimate the distance to prey (Rossel 1983; Nityananda et al. 2016). A prerequisite for this behaviour is neuronal machinery that takes into account the slightly shifted images both eyes see of the same region in space in front of the animal. From the amount of the shift—the disparity—between the two eyes’ views, the distance of objects can then be triangulated. Neurons suited to do this computation are called disparity sensitive*.* They have been found in the visual cortices of cats and monkeys (Cumming and DeAngelis 2001; Parker 2007) and more recently in the praying mantis brain (Rosner et al. 2019). So far, four neuron types have been described that are tuned to specific retinal disparities. All neurons ramify in one or both optic lobes and together they cover a range of processing stages. One columnar type of neuron, *COcom*, ramifies in both optic lobes, specifically in both lobula complexes. Anatomically COcom neurons seem most suited to perform the initial combination of binocular information, a prerequisite for disparity sensitivity, in the praying mantis brain because they could either combine binocular information directly or deliver monocular information from one brain hemisphere to the opposite side. A tangential type of projection neuron, *TAOpro*, ramifies in one lobula complex only and presumably delivers the signal to descending neurons. Members of the COcom neuron class as well as the tangential projection neuron were shown to be tuned to specific locations in 3D space that are within the catching range of the animals. Thus, these neurons could be responsible for mediating the raptorial strike when prey is in reach. The two other classes of neuron are presumably further downstream. They are centrifugal and thus convey disparity information in the opposite direction from the central brain to the optic lobes. The purpose of this centrifugal pathway is unknown but could include, for example, allocation of spatial attention in 3D.

In order to more thoroughly understand the extent of binocular vs monocular processing in the praying mantis optic lobe we identified output neurons of the optic lobe by tracer injections with sharp electrodes and determined the frontal binocular response fields of these neurons. We report all neurons we encountered, those which appear to respond to visual input from only one eye (monocular, 7/19) and those which respond to both (binocular, 12/19).

## Materials and methods

Experimental procedures were largely the same as for Rosner et al. (2019). We reproduce these methods here with only slight adjustments.

### Animals

The experiments were carried out on 19 large adult praying mantids. Animals were purchased from BugzUK (Norwich, UK), M&M Wüst (Mühlheim am Main, Germany) and Mantidendealer (Berlin, Germany). Supply limitations meant we used both male and female individuals of three closely related species: *Rhombodera megaera*, *Hierodula membranacea* and a slightly smaller species of the genus *Hierodula*, presumably *Hierodula unimaculata* (Table 1). These species all belong to the same tribe, *Paramantini,* of the *Mantidae* family. We assigned the animals to two size classes with an interocular distance of either 8 mm or 6 mm. Insects were housed in individual containers at a temperature of 25 °C and a 12 h light/dark cycle. Adult animals were fed with a live cricket twice and younger mantids three times a week.

**Table 1:** Properties of all recorded projection neurons of the optic lobe

Neuron class	Neuron ID	Presumed input	Presumed output	Stimulus contrast	Figure	Repetitions	ANOVA	*p *values (L/R/L × R)	Variance explained by fitted model	IOD	Species	Sex
TOpro1	rr160420	OLO1 (II), 2 (I, II)	VMNP,VLNP,INP	Dark	2b2	11	L/ns/ns	0.000/ 0.429/ 0.659	68%	8	H.m	F
TOpro1	rr160125	OLO1 (II), 2 (I,II)	VMNP,VLNP,INP	Dark	2a2	35	L/ns/ns	0.000/0.686/0.884	86%	8	H.m	F
				Bright	2a3	29	L/ns/ns	0.000/0.140/0.632	91%			
TOpro2	rr160301	OLO1 (II), 2 (I)	VLNP,INP	Dark	2c2	10	L/ns/ns	0.000/0.372/0.562	83%	8	H.m	F
TOpro3	rr160503	OLO1 (II), 2 (I,II)	VLNP	Dark	2d2	10 inj (0.5 nA)	L/R/I	0.000/0.000/0.016	47%	8	H.m	F
TOproX	rr170601	OLO1 (II), 2 (II)	Central brain	Dark	2e2	17	L/ns/ns	0.001/0.888/0.979	56%	8	H.m	F
TAprodistX (probably TADpro)	rr170404	ALO-V (I or II distal surface)	Uncertain	Dark	3a3	10	L/R/ns	0.000/0.008/0.660	71%	8	H.m	M
				Bright	3a2	10	L/R/I	0.000/0.000/0.001	88%			
TApro_dist_X	rr160202	ALO-V (I, II surface)	presumably VMNP	Bright	3f2	18	L/R/ns	0.000/0.000/0.164	98%	8	H.m	F
TApro_dist_1	rr151111	ALO-V (I, II surface)	VMNP	Bright	3e2	16	L/R/I	0.000/0.000/0.000	97%	8	H.m	F
TADpro	rr161115	ALO-V (I, II surface), DLO	VMNP (contral.), VLNP (contral.)	Dark	3b3	10	ns/R/ns	0.062/0.000/0.271	74%	6	H.u	F
				Bright	3b2	12	L/R/I	0.000/0.000/0.000	92%			
TADpro	rr160309	ALO-V (I, II surface), DLO	VMNP (contral.), VLNP (contral.)	Dark	3c3	13	L/R/I	0.000/0.000/0.000	92%	8	H.m	F
				Bright	3c2	9	L/R/I	0.000/0.000/0.043	98%			
TADpro	rr161102	ALO-V (I, II surface), DLO	VMNP (contral.), VLNP (contral.)	Dark	3d2	10	ns/R/ns	0.774/0.000/0.094	36%	8	R.m	M
TApro_prox_1	rr151013	ALO-V (III/IV)	VLNP, VMNP	Dark	4a2	10	L/R/I	0.000/0.000/0.003	81%	8	H.m	F
TApro_prox_1	rr170124	ALO-V (III/IV)	VLNP, VMNP	Dark	4b2	12	L/R/ns	0.000/0.000/0.873	99%	8	R.m	M
TApro_prox_2	rr151102	ALO-V (III/IV)	VLNP, VMNP	Dark	4c2	26	L/ns/ns	0.000/0.848/0.430	94%	8	H.m	F
TApro_prox_2	rr170125	ALO-V (III/IV)	VLNP, VMNP	Dark	4d2	10	L/R/ns	0.000/0.000/0.272	92%	8	R.m	M
TApro_prox_3	rr170629	ALO-V (III/IV)	VMNP, VLNP	Dark	4e2	10	L/R/ns	0.000/0.000/0.759	92%	8	R.m	M
Spro	rr170203	SLO	VLNP, INP	Dark	4f2	12	L/R/ns	0.000/0.010/1.000	78%	6	H.u	F
TMeASpro	rr170605	Medulla	ALO-V (proximal), ALO-D (proximal), SLO, optic stalk, SNP	Dark	5b2	14	L/ns/ns	0.000/0.113/0.060	61%	8	H.m	F
TMeOSpro	rr160216	Medulla, OLO1 (II), 2 (I, II), SLO	VLNP, INP	Dark	5a2	17	L/R/I	0.000/0.000/0.000	90%	8	H.m	F

We provide preliminary neuron names in column 1. The nomenclature mainly follows the one suggested by Rosner et al. (2017). *TOpro* tangential projection neuron of the outer lobes, *TApro* tangential projection neuron of the anterior lobe, *TADpro* tangential projection neuron of the anterior lobe and the dorsal lobe, *TMeASpro* tangential projection neuron of the medulla, the anterior lobe and the stalk lobe, *TMeOSpro* tangential projection neuron of the medulla, the outer lobes and the stalk lobe. To highlight whether a particular TApro neuron ramifies in distal or proximal layers we added “prox” or “dist” and neurons that could not be unequivocally identified were provided an “X” instead of a number. Neurons with distal ALO ramifications seemed to possess two tangential fans. We assume that one of them ramifies in layer 1 and the other potentially at the surface of layer 2. Neurons with ramifications in ALO-V layer 3 (Rosner et al. 2017; Yamawaki 2019) sometimes had two fans and we assume that layer 3 actually consists of two layers of which we call the most proximal one layer 4

The eighth column indicates whether there was a significant (*p* < 0.05) main effect of left (L) or/and right (R) eye stimulation on neuronal response as tested with two-way-ANOVA. A significant interaction term in the two-way-ANOVA is indicated by “I”, non-significance by ‘ns’. *p* values are provided in 9th column. *p* values smaller than 0.0001 are given as 0.000. The 10th column gives the percentage of variance explained by the fitted model

Recordings were usually done without concurrent depolarizing current injection unless indicated by ‘inj’ with the amount of current indicated in nA. Lobula complex neuropil abbreviations: *ALO-V* ventral lobe of the anterior lobe, *DLO* dorsal lobe, *OLO1/2* outer lobes 1/2, *SLO* stalk lobe. Layers with ramifications are given in brackets. The layers with the smallest number are more distal than layers with larger numbers. Layer assignments in the ALO are preliminary. We provide the central brain output regions as neuropil supercategrories as described by Ito et al. (2014)

*INP* inferior neuropils, *SNP* superior neuropils, *VLNP* ventrolateral neuropils, *VMNP* ventromedial neuropils. Additional abbreviations: *H.m.Hierodula membranacea*, *H.u.Hierodula unimaculata*, *L* left eye, *I* interaction (L × R), *IOD* interocular distance, *ns* not significant (*p* > 0.05), *R* right eye, *R.m.Rhombodera megaera*

### Animal preparation

Animals were mounted on custom-made holders with BluTack® and wax; their mouthparts were removed, and their head was immobilized by wax. A hole was cut into the posterior head capsule to allow access to the brain. Fat and muscle tissue surrounding the brain were removed. The neural sheath was stripped away at the region where the recording electrode was inserted. The gut was removed within the head capsule and prevented from leaking within the thorax by ligating it. A wire platform supported the brain from anterior to further stabilize it. During recording of neural activity the brain was submerged in cockroach saline.

### Neuronal recordings

All recordings were performed exclusively in the left optic lobe. We expect the same set of neurons is present on both sides of the brain. We recorded intracellularly with sharp electrodes from 19 neurons. Each cell was recorded in a different animal. We gained stainings of all 19 neurons even though in some cases the stainings were not complete. When more than a single neuron was stained during the course of one experiment we assumed that the cell with the strongest staining was the one we recorded from. With the here applied technique of intracellular recordings, multiple neuron stainings can occur by leakage of tracer from the micropipette when trying to establish a stable recording. All neurons are listed in Table 1.

We presume that these cells receive their main sensory input in the optic lobe because of the proximity of these ramifications to their respective cell body. An additional indicator for the cells’ polarities were smooth ramifications in the optic lobe and beaded ramifications in the central brain even though this distinction was not possible for all cells. Dendritic morphology as well as proximity of the soma to terminal neurites is a good predictor for input and output structures of insect neurons (Grueber et al. 2005; Cardona et al. 2010). All neurons had ramifications in the LOX and two had additional ramifications in the medulla. The neurons were identified by stainings with neuronal tracer (see below). Microelectrodes were drawn from borosilicate capillaries (1.5 mm outer diameter, Hilgenberg, Malsfeld, Germany) on a microelectrode puller (P-97, Sutter Instrument, Novato, CA). Electrode tips were filled with 4% Neurobiotin (Vector Laboratories, UK) in 1 M KCl and their shanks with 1 M KCl. The electrodes had tip resistances of 70–150 MOhm. Signals were amplified (BA-03X amplifier; NPI), digitized (CED1401 micro; Cambridge Electronic Design, UK) and stored using a PC with Spike2 software (Cambridge Electronic Design, UK). About 0.1–1 nA of depolarizing current was applied for several minutes to iontophoretically inject Neurobiotin immediately after visual stimulation and in some recordings in-between the stimulus sequences. We only injected and analysed those neurons for which we could acquire responses to the presentation of at least nine repetitions of the bar stimulus.

### Histology

After neuronal recordings animal heads were fixed overnight in a mixture of 4% paraformaldehyde, 0.25% glutaraldehyde, and 0.2% saturated picric acid in 0.1 M phosphate buffer. Afterwards brains were dissected out of the head capsule. The labelled neurons were made visible for confocal laser scanning microscopy (Leica TCS-SP5/SP8; Leica Microsystems) by treatment of the brains with Cy3-conjugated streptavidin (Dianova, Hamburg, Germany). More specifically after incubation with the fixative, brains were first washed with 0.1 M PBS and then with 0.1 M PBS containing 0.3% Triton X-100. Afterwards the brains were incubated with streptavidin-Cy3 for 3 days at 4 °C. Then the brains were again washed in PBS before dehydrating them in an ethanol series (25, 50, 70, 90, 95, and 100%, 15 min each). Finally, the brains were cleared by first treating them with a solution of 50% ethanol and 50% methyl salicylate (20 min) and then with pure methyl salicylate (Merck, Darmstadt, Germany) until transparent (at least 60 min). As a last step the brains were mounted in Permount (Fisher Scientific, Pittsburgh, PA) between two glass cover slips which were separated by spacing rings to avoid compression.

### Visual stimulation

**Fig. 1:**
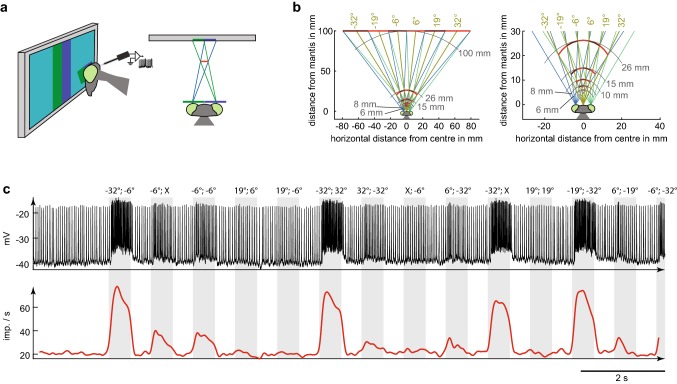
Experimental setup and vertical bar stimulus. **a** A praying mantis, equipped with a green and a blue spectral filter, watches the flashed bar stimulus on a computer screen. The illustrations show the screen displaying two vertical bars, one for each eye, near the centre of the screen (side view, top view). The lines of sight are shown in blue (green) for the left (right) eye. This creates the same retinal images as a virtual bar floating in front of screen (red line). **b** Diagrams explaining the bar stimulus. Bars were presented at 6 different, non-overlapping locations on the computer screen. Bar centres were at − 32°, − 19°, − 6°, 6°, 19° and 32°with regard to the centre of the mantis head. Negative (positive) values indicate left (right) side. The computer screen was 100 mm distant from the animal. Bars (including virtual/simulated bars) are shown alternating dark/bright red for clarity (right panel zoom). Azimuthal direction of simulated bars from the mantis head midline are shown in olive and distance isolines in grey. The figure is adapted from Rosner et al. 2019. **c** Upper panel shows recording section from experiment rr170124 during dark bar stimulation (for response field see Fig. 4b2). Lower panel shows spiking rate estimated by Gaussian filtering with SD of 50 ms. Vertical, grey shaded areas indicate times of bar display. The left and right eye bar locations are given on top (left eye left number, right eye right number). X means no bar was shown for this eye

We used anaglyph technology (Nityananda et al. 2016) to present 3D stimuli on a computer monitor (DELL U2413 LED, 60 Hz). Tethered mantids watched the computer screen through spectral filters while we performed neuronal recordings in their brain (Fig. 1a). We presented stimuli with different colours (green and blue) that matched the spectral properties of the filters so that each eye saw only the image it was intended to see. We performed electroretinograms as described in Nityananda et al. (2016) on an additional set of animals to ensure same perceived brightness through both spectral filters by adjusting the brightness gain for both colour channels accordingly. The computer screen was positioned at a viewing distance of 10 cm from the praying mantis.

The stimulus was custom written in Matlab (Mathworks.com) using the Psychophysics Toolbox (Brainard and Vision 1997; Pelli 1997; Kleiner et al. 2007). We analysed monocular and binocular response fields of neurons with a flashed bar stimulus. For this we divided the region of binocular overlap into six non-overlapping vertical stripes of 12.8° horizontal and 99.5° vertical extent (Fig. 1b). In this way we covered almost 77° of the fronto-azimuthal visual field. This is slightly wider than the approximately 70° binocular overlap of praying mantids (Rossel 1986). Bars were presented either to one eye only, for recording monocular response fields, or two bars concurrently, one for the left and one for the right eye, for determining binocular response fields. This means that there were 48 conditions for each repetition or trial. This number is the sum of 12 monocular bar presentations (6 bar locations for the left eye and the same 6 locations when stimulating the right eye only). Additionally to these 12 monocular presentations, all combinations of the six bar locations for the left and right eye were presented. These were 36 binocular presentations.

We used bars instead of structures with smaller vertical extent because of the comparatively short recording times possible with sharp electrodes. In this way we avoided the need to identify receptive field elevation while enabling us to vary horizontal disparity, the difference in the bar’s location between left and right eyes. Because insect eyes are offset horizontally and fixed on the head, horizontal disparity along with visual direction specifies a unique location in the *x*–*z*-plane (Rossel et al. 1992; Erkelens and van Ee 1998), as shown in Fig. 1b. All bar combinations, including both monocular and binocular conditions, were shown in pseudorandom order; we refer to this as one repetition. The bars were displayed for 500 ms with a pause of the same duration in between each presentation. In one recording (experiment rr170629) the bars were displayed for 250 ms with pauses of 300 ms in between. After all bar positions had been displayed a pause of 1.7–4.5 s followed, before the procedure started again. These stimulation times and pauses were chosen after preliminary experiments had shown that they sufficed our requirements for (1) being long enough to elicit strong responses and thus reliable response estimates, (2) not influencing successive stimulations and (3) still providing sufficient time to acquire at least nine repetitions with at least one bar condition (providing dark or bright bars).

We presented dark or bright bars in front of a grey background but never bright and dark bars at the same time. The grey background had the average luminance of the dark and bright bars. We aimed at presenting a full set of at least nine repetitions of the full sequence of bar positions for both stimulus contrasts, but in most experiments this was not possible because of the limited time available during intracellular recordings. In order to use the recording time most efficiently we determined the preference of a neuron for bright or dark stimuli by showing bright on and off flashes of the computer screen; however, in several cases we just started with a dark bar stimulus because originally we were interested in finding prey detector neurons and mantids prefer dark prey in front of a bright background. If the cells did not show prominent responses to the dark bar stimulus we switched to the bright bar stimulus instead before a set of nine repetitions of all dark bar stimulation had been acquired, in order to increase the likelihood of acquiring a full set of nine repetitions with an effective stimulus.

### Microscopy and image data analysis

Whole mounts were scanned with confocal laser scanning microscopes (CLSM, TCS SP5 and SP8, Leica Microsystems, Wetzlar, Germany) with a 10 × oil immersion objective lens (SP5) or a 10 × dry lens (SP8). The SP5 microscope was located in the Biology Department of Marburg University (Germany) and the SP8 microscope in the Bioimaging Unit at Newcastle University (UK).

Neuronal reconstructions were done with Imaris 9.3.1 (Bitplane, Belfast, UK). If more than a single neuron was stained we reconstructed the one which was stained most strongly. For visualising the location of neurons in the praying mantis’ brains we superimposed the neurons on schemes of mantis brains and adjusted the shape of the schematised brain and in some cases also neuropils to better capture individual differences between animals and distortions of the brains caused by the preparation procedure after the electrophysiological experiments. The schemes of the mantis brains were modified versions of those used in Rosner et al. (2019) and the registration of the neurons onto these schemes was done in Adobe Illustrator CC 2018 (Adobe Systems, Ireland).

### Data evaluation

Data analysis was done in Matlab (The MathWorks, Natick, MA) and is based exclusively on spike counts.

Bar stimulus-induced spike counts were determined in 250 ms time windows starting at time 1 ms when a bar was displayed. The background spike count was determined in 800 ms time windows preceding each stimulus repetition. We found that neurons’ responsiveness could fluctuate over time such that the same sequence might elicit higher or lower spike rates depending on when it was presented. To minimise the effect of this fluctuation, we normalized responses by the highest spike count observed during that repetition. This could be the spike count for one of the bar presentations or alternatively it could be the background spike count. All spike counts in a given repetition were divided by the highest spike count observed for that repetition to produce normalized responses. Afterwards, these normalized responses were averaged across all repetitions of the same stimulus for each cell, to produce the mean normalized responses shown in the figures.

For presentation purposes, we interpolated all binocular response fields from 6 × 6 to 100 × 100 with the Matlab function imresize in bicubic mode. This performs convolution-based interpolation according to the algorithm in Keys (1981).

### Statistical analysis

Responsiveness of neurons to left or right eye stimulation was determined by two-way-ANOVA (anova2-function in Matlab; requirement for significance *p* < 0.05). The two factors were the location of the bar in the left and right eye, respectively. Each factor had seven levels, corresponding to the six possible bar locations plus the blank-screen condition. A significant main effect of each factor, therefore, means that the response differed between at least two different bar positions for the respective eye, and/or the response differed for at least one bar location from the spontaneous rate. A non-significant interaction term means that binocular response was well described by the sum of monocular responses; a significant interaction means that they combine non-linearly. We call a cell “binocular” for a particular stimulus if the left and right eye factors both have significant main effects, and/or if their interaction was significant (see Table 1). Since ANOVA requires the residuals to be normally distributed, which was not always the case, we also confirmed binocularity using the non-parametric Friedman test.

### Modelling

For simulating response fields we applied a “linear/non-linear” (LN) model used for modelling simple cell responses in vertebrate stereopsis (the simple cell model in (Ohzawa et al. 1990), generalised to allow arbitrary receptive fields and output exponent) and insect stereopsis (Rosner et al. 2019). This model assumes that visual stimulation contributes excitatory or inhibitory input dependent on the eye and location of the stimulation, that is, the model contains receptive fields for both the left and the right eye. The inputs from both eyes are filtered by the receptive field and then summed linearly along with a tonic input, necessary to account for a non-zero background rate in some neurons. If the result is negative or zero, the mean response is zero (threshold nonlinearity). If the result is positive, the mean response is given by its value raised to some exponent (power-law nonlinearity). Note that we use the term response field to mean the measured average spiking rate of the neuron to bar stimuli at the specified location; we keep the term receptive field to refer to the linear part of the function governing this response, which is not directly observable. The fitting procedure was exactly the same as in Rosner et al. (2019). Specifically, we fitted the model to the mean neuronal response, that is, to the normalized spike count, *D*_*ij*_, where *i*, *j* indexes the stimulus present in left, right eyes. The six bar positions are indexed by *i* = 1,…, 6, and we use *i* = 0 to indicate that no bar was present. The full model has 14 parameters: *L*_*i*_, *R*_*i*_ represent the response of the left, right eye receptive field to a bar at position *i* (*i* = 1,…,6) in that eye; *b* is the tonic input; and *γ* is the exponent of the output non-linearity. These 14 parameters are fitted to the mean neuronal response in 49 conditions (no visual stimulation, 12 monocular conditions and 36 binocular), so as to make the model response *M*_*ij*_ as close to the observed *D*_*i*j_ as possible.

The model response *M*_*ij*_ is thus as follows: The background response of the model neuron in the absence of visual stimulus is

$$ M_{00} = b^{\gamma } ; $$

the response to a monocular bar at the *i*th position in the left, right eye is

$$ M_{i0} = \left\lfloor {L_{i} + b} \right\rfloor^{\gamma } ,\;M_{0i} = \left\lfloor {R_{i} + b} \right\rfloor^{\gamma } ; $$

and the response to binocular bars at the *i*th position in the left eye and the *j*th position in the right eye is

$$ M_{ij} = \left\lfloor {L_{i} + R_{j} + b} \right\rfloor^{\gamma } , $$

where $\left\lfloor x \right\rfloor = x$ if x > 0 and 0 otherwise (i.e. a threshold at 0). The sum of squared errors between model and data is

$$ \varepsilon = \sum\nolimits_{i = 0}^{6} {\sum\nolimits_{j = 0}^{6} {\left( {D_{ij} - M_{ij} } \right)} }^{2} . $$

We also included a regularisation term Λ intended to keep parameter values close to zero except where they clearly improved the fit. This term was equal to one-thousandth of the summed squared parameters:

$$ \Lambda = 0.001\;\left[ {b^{2} + \gamma^{2} + \sum\nolimits_{i = 1}^{6} {\left( {L_{i}^{2} + R_{i}^{2} } \right)} } \right] $$

The 13 parameters *L*_*i*_, *R*_*i*_ and *γ* were adjusted so as to minimise *ε* + *Λ* across all data. The parameter *b* was not fitted as a free parameter, but was constrained so as to account for the background firing rate of the cell given the fitted output exponent *γ*, i.e. we set

$$ b = D_{00}^{(1/\gamma )} $$

In practice, this constraint makes little difference compared to fitting all 14 parameters together freely.

Fitting was carried out by the Matlab routine FMINSEARCH. Convergence to local optima can be a problem in such multi-parameter optimisation, and the choice of initialisation is often critical. We started by doing a 12-parameter fit with *γ* set to 1 and *b* set to *D*_00_. We explored two initialisations: a flat initialisation (*L*_*i*_ = 1, *R*_*i*_ = 1 for all *i*), and an initialisation reflecting the monocular responses (*L*_*i*_ = *D*_*i*0_–*D*_00_, *R*_*i*_ = *D*_*0i*_*–D*_00_), and selected whichever gave the lowest fit error *ε* + *Λ*. We then removed the constraint on *γ* and performed the full 13-parameter fit. We again explored two initialisations: the *L*_*i*_*,R*_*i*_ found by the 12-parameter fit, and the monocular-response initialisation *L*_*i*_ = *D*_*i*0_*–D*_00_,* R*_*i*_ = *D*_0*i*_*–D*_00_, both with *γ* = 1, and again chose whichever yielded the lowest fit error. We found that with these methods, the optimisation converged rapidly and reliably.

To compute percentage of variance explained, we computed the total variance of the mean observed response in each stimulus condition:

$$ T = \frac{1}{48}\sum\nolimits_{i = 0}^{6} {\sum\nolimits_{j = 0}^{6} {\left( {D_{ij} - \overline{D}} \right)^{2} } } $$

and the residual variance of the difference between the mean observed response and the model fit in each stimulus condition:

$$ R = \frac{1}{48}\sum\nolimits_{i = 0}^{6} {\sum\nolimits_{j = 0}^{6} {\left( {D_{ij} - M_{ij} - \overline{D} + \overline{M}} \right)^{2} } } ; $$

the percentage of variance explained is then ${\text{PV}}\; = \;100\;\left( {T - R} \right)/T$.

## Results

We recorded from 19 projection neurons with presumed input ramifications in the optic lobe and output in the central brain. We used a vertical bar visual stimulus in order to map the neurons’ frontal visual responses in the region of binocular overlap. 12 of the 19 tested neurons were demonstrated to be binocular for either dark or bright bars or both. However, we did not test all neurons with both contrasts due to the limited period of time available when doing intracellular recordings.

### Neurons of the outer lobes

We recorded from five different projection neurons with input in the most distal neuropils of the praying mantis lobula complex (LOX): the outer lobe 1 (OLO1) and the outer lobe 2 (OLO2). Two of the cells belonged to the same cell type, *TOpro1* (tangential projection neuron of the outer lobes; (Rosner et al. 2017)) also called L7-cell by (Berger 1985) or TOproM1 by (Yamawaki 2019) to additionally highlight its output ramifications within the ventromedial neuropil (Ito et al. 2014) in the ipsilateral central brain. The remaining three neurons are listed in Table 1 as TOpro2, TOpro3 and TOproX and probably belong to a group of cells which had earlier been summarized as L4-cells by Berger (1985). All neurons ramified in OLO1 as well as in OLO2.

**Fig. 2:**
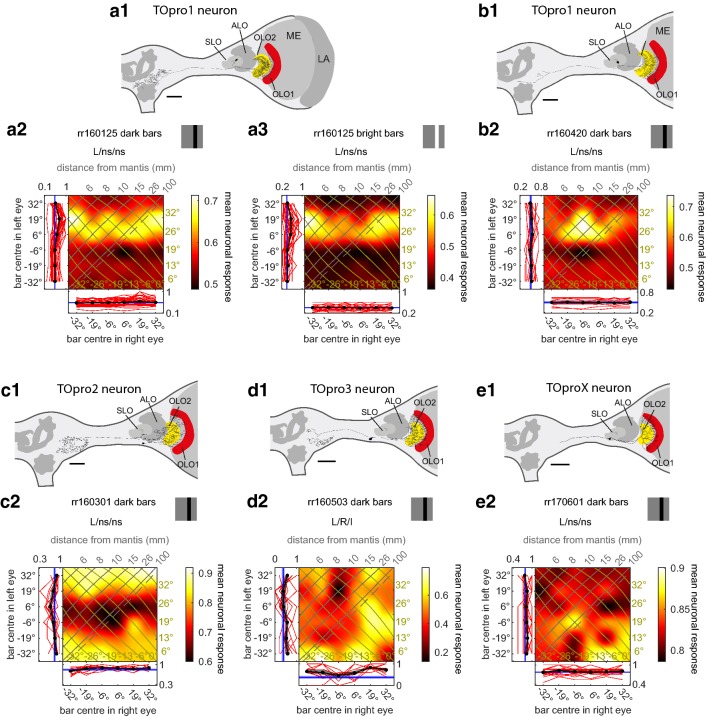
Responses of projection neurons with input in the outer lobes of the lobula complex. **a1**, **b1**, **c1**, **d1**, **e1**, Frontal views of reconstructions of tangential neurons of the outer lobes of the LOX. The reconstructed neurons are superimposed on the scheme of the left hemisphere of a praying mantis brain with key neuropils shown. The shape of the brains and of the outer lobes was adjusted to better capture the location of the neurons within the brain and the neuropils. All scale bars are 200 µm. ALO, anterior lobe; LA, lamina; ME, medulla; OLO1/2, outer lobes 1/2; SLO stalk lobe. **a1**, **b1** Reconstructions of TOpro1 neurons from experiments rr160125 and rr160420 with ramifications in OLO1 (red) and both OLO2 (yellow). The brain with the TOpro1 neuron shown in b1 was damaged when it was extracted from the head capsule. Thus, the reconstruction consists of two parts. **a2**, **a3** Monocular and binocular responses of TOpro1 neuron rr160125 to dark (**a2**) and bright (**a3**) bar stimulus. Binocular responses as pseudocolour 2D-plot and monocular responses (individual responses red lines; averages ± 1 SEM black line; blue line background activity) as 1D-plots at left and bottom margins for respective eye. Axes show centre of bar shown to left and right eye, respectively. The binocular response is interpolated; cf Supp Fig. 2 of Rosner et al 2019. Isolines mark azimuth (olive) and distance (grey) from the mantis head as shown in Fig. 1b. Dashed line marks screen locations implying infinitely distant objects, i.e. where the lines of sight to each eye are parallel. Neuronal responses are normalised to the highest spike count that occurred during either bar presentation or background firing, depending which one was higher (see Methods). The header states the code assigned to the individual neuron and whether ANOVA indicated a significant main effect for the left (L) or right (R) eye and whether there was a significant interaction term (I). A non-significant response is indicated with “ns”. Thus, for this neuron, there was a significant main effect for the left eye only. **b2**, **c2**, **d2**, **e2** Monocular and binocular responses of the four further TOpro neurons to dark bar stimulation. For projection views of reconstructed neurons see Suppl. Fig. 1

TOpro1 had the most complex dendritic tree with three big fans carrying dendritic ramifications for both layers of OLO2 and the more proximal of the two layers of OLO1 [Fig. 2a1, b1, (Rosner et al. 2017)]. The soma of TOpro1 was located in the frontomedial soma rind and the neurite carrying the soma travelled through the hole of the tunnel-shaped stalk lobe. The somas of the remaining three neurons were located more ventrally in the optic lobe and two of the neurons had only two dendritic fans, one ramifying in OLO1 and the other in OLO2 (Fig. 2d1, e1). The exact locations and morphology of the fans within the outer lobes differed between all three neurons. Two of the three cells had their output ramifications in the ipsilateral ventrolateral neuropil but differed with regard to their specific layout and location (compare Fig. 2c1, d1). For one neuron the central brain ramifications could not be traced due to incomplete staining (Fig. 2e1). Because the identity of this neuron cannot be determined with certainty, we call it TOproX in Table 1.

Physiologically all but one of the five cells were monocular (received input from only one eye, as indicated statistically by a significant main effect of bar position only in that eye and no significant interaction term). These all had their input from the left eye, the side of all of our recordings and also the side of all input and output ramification. Both TOpro1 neurons were monocular for the dark bar stimulus (Fig. 2a2, b2). Their interpolated binocular response fields suggest that the most effective location for eliciting excitatory responses is in the left eye, from 6° and 19° to the right, that is the contralateral side, of the animals. The response fields for the TOpro1 neuron in Fig. 2a1 are typical for a monocular neuron. The horizontal stripe in the binocular response field indicates that this neuron responded to the location of the stimulus when presented to the left eye and is largely unaffected by the presence or location of a bar shown concurrently to the right eye (cf Fig. 6a). Monocular neurons like this cannot be tuned to stereoscopic distance. Conversely, a binocular neuron which is tuned to a single specific distance and azimuthal location will respond best to a certain combination of a left and right eye bar. Thus, the binocular response field of such a neuron will tend to show a patch instead of a stripe, as we see later (e.g. Fig. 5a2, Fig. 6b–h).

One of the TOpro1 neurons was also tested with bright bars and bright flashes of the whole computer screen. Even though the cell responded with phasic excitations only to the offset of the whole screen flash it did respond to the bright bars with a very similar response field as for the dark bar stimulus (compare Fig. 2a2 and a3). A clear stripe was again visible in the binocular response field, as expected for monocular neurons.

Another cell (Fig. 2c1), which we call TOpro2, was also monocular, with a clear stripe in the binocular response field (Fig. 2c2). This neuron was inhibited by dark bars in the left eye with a bar centre at either 6° to the left or 6° to the right. And two further TOpro neurons (Fig. 2d1, e1) had rather complex frontal binocular response fields when stimulated with dark bars (Fig. 2d2, e2). One of these two neurons was binocular (Fig. 2d2, Table 1) but was not tuned to a single azimuthal location and distance (no single patch in the binocular response field).

### Neurons of the anterior lobe

We recorded from 11 projection neurons with their main input ramifications in the anterior lobe (ALO) of the LOX. The ALO consists of a ventral (ALO-V) and a dorsal (ALO-D) subunit and both units can be further subdivided into several layers. Most relevant for the current study is the division of ALO-V into at least three layers (Rosner et al. 2017; Yamawaki 2019). However, from our stainings of individual neurons and from screening the background stainings of all brains of the current study and those from Rosner et al. (2019) we suspect that ALO-V will prove to contain a total of four layers (data not shown). In Table 1 we provide a preliminary assessment of which layers are innervated by the neurons.

**Fig. 3:**
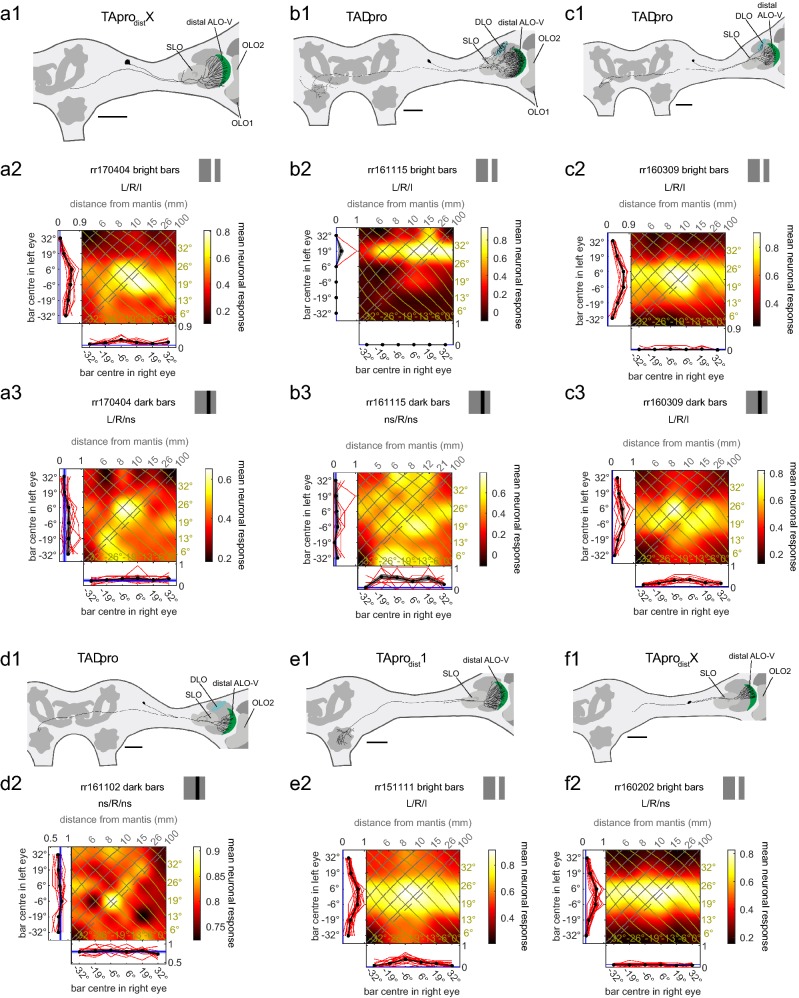
Responses of tangential projection neurons with input in the distal anterior lobe of the lobula complex. **a1**, **b1**, **c1**, **d1**, **e1** Frontal views of reconstructions of tangential neurons of the distal anterior lobe (coloured) of the LOX. Some neurons were not completely stained (**a1**, **c1**, **d1**, **f1**). However, **c1** and **d1** were stained well enough to assign them to the same type as the neuron in **b1**. The neuron in **a1** also presumably belongs to this type (see main text). As in Fig. 2 the reconstructed neurons are superimposed on the scheme of relevant parts of a praying mantis brain with key neuropils shown. The shape of the brains was adjusted to better capture the location of the neurons within the brain and the neuropils. All scale bars are 200 µm. *ALO-V* ventral lobe of the anterior lobe, *OLO1/2* outer lobes 1/2, *SLO* stalk lobe. The neuron in **f1** presumably belonged to the same type as the neuron in **e1**. The neuron was not completely stained in the central brain. Additionally, it was co-stained with another neuron in the distal ALO and we did not reconstruct potentially existing ventral ramifications because we could not easily distinguish them from neurites of the other neuron. We cautiously name the neuron in **f** TApro_dist_X with X standing for unknown type. **a2**, **a3**, **b2**, **b3**, **c2**, **c3**, **d2**, **e2**, **f2** Monocular and binocular responses of the neurons in **a1**, **b1**, **c1**, **d1**, **e1**, **f1** to the stimulus conditions stated in the headers of the plots. For projection views of reconstructed neurons see Suppl. Fig. 2

**Fig. 4:**
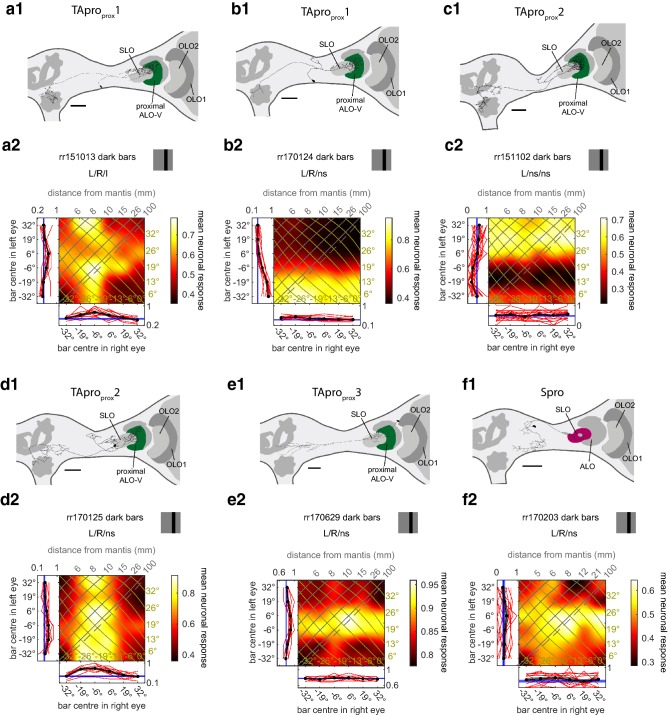
Responses of projection neurons with input in the proximal anterior lobe and the stalk lobe of the lobula complex. **a1**, **b1**, **c1**, **de1**, **e1**, **f1** Frontal views of reconstructions of neurons with input ramifications in the proximal anterior lobe (green) or the stalk lobe (purple) of the LOX. As in Fig. 2 the reconstructed neurons are superimposed on the scheme of relevant parts of a praying mantis brain with key neuropils shown. The shape of the brains was adjusted to better capture the location of the neurons within the brain and the neuropils. The LOX neuropil with ramifications is coloured. Note that only the neuron in **f** ramifies in SLO; ramifications which may appear to terminate in SLO in the other 2D images are not in the same depth as SLO. All scale bars are 200 µm. *ALO-V* ventral lobe of the anterior lobe, *OLO1/2* outer lobes 1/2, *SLO* stalk lobe. **a2**, **b2**, **c2**, **de2**, **e2**, **f2** Monocular and binocular responses of the neurons in **a1**, **b1**, **c1**, **de1**, **e1**, **f1** to the stimulus conditions stated in the headers of the plots. For projection views of reconstructed neurons see Suppl. Figure 3

Physiologically there was one striking difference between neurons with their main input in the distal ALO-V layers 1 and 2 (Fig. 3) and neurons with their main input in the proximal ALO-V layers 3 and 4 (Fig. 4). ALO neurons with distal ramifications were most sensitive to bright screen flashes (Suppl. Figure 5) and showed the clearest disparity tuning to bright bars (e.g. Fig. 3b2 vs b3). In contrast, neurons with their main input in the proximal ALO-V responded more vigorously to the offset of screen flashes and to dark bars (Suppl. Fig. 6). Nine of the 11 ALO neurons were binocular for either dark or bright bars or both, indicating that they receive input from both eyes in the frontal region of binocular overlap.

#### Neurons with ramifications in distal anterior lobe layers

Six of the neurons received their input via fan-shaped dendritic trees in distal ALO-V layers (Fig. 3). Five of the cells were tested with bright bars and their responses were very similar to each other. The neurons were all binocular for this stimulus and the binocular response fields show an excitatory, horizontal stripe indicating a dominant left eye (compare Fig. 3a2, b2, c2, e2, f2). At least three of the cells (Fig. 3b–d) belonged to a cell type called TAproM2 by Yamawaki (2019) because of its tangential ramifications in the ALO and its output within the contralateral ventromedial neuropils (VMNP). The same neuron was called L15 by Berger (1985). We call this type TADpro because of its additional prominent ramifications in the dorsal lobe (DLO) of the LOX. A fourth cell (Fig. 3a1) presumably belonged to the same type; however, it could not unequivocally be identified because of its weak staining. The axon could not be completely traced and neither could the DLO ramifications; however, the ALO ramifications, the soma location and the axon path suggested that it was a TADpro neuron. We call it TApro_dist_X in Table 1 because of its uncertain identity. One neuron (Fig. 3e) had very similar input ramifications in the distal ALO-V but lacked prominent DLO ramifications. Moreover, its output was in the ipsilateral VMPN. It could be the neuron called TAproM1 by Yamawaki (2019) and L9 by Berger (1985). However, there were morphological differences like missing ramifications in the DLO and differences with regard to the neurite carrying the soma. The soma was lost during preparation after the electrophysiological experiment. We call the cell TApro_dist_1 in Table 1. Finally, one neuron with again similar ramifications in the distal ALO-V was weakly stained within the central brain and overlapped with at least one additional neuron with ramifications also in the distal ALO-V. We think it is likely that it was of the same type as the TApro_dist_1 cell but cautiously call it TApro_dist_X in Table 1. This cell also had a similar response profile to the other ALO-V neurons with distal ramifications (Fig. 3f1, f2).

Five of the six neurons with distal ALO ramifications were tested with bright bars and all of the neurons showed a dominant excitatory input from the ipsilateral (left) eye as seen by a clear horizontal stripe in the binocular response fields (Fig. 3a2, b2, c2, e2, f2). However, 2-way ANOVA also indicated a significant main effect of right-eye stimulation for all five cells (this was confirmed by the non-parametric Friedman test for all except one neuron, Fig. 3f). The highest spiking rates occurred for bars at simulated distances between 26 mm to infinity and for some cells even beyond this for diverging lines of sight (e.g. Fig. 3a2, where strong response is elicited when bars are presented at − 6° in the left eye and + 6° in the right, which does not correspond to the location of any single object in space).

We tested three neurons additionally and a single neuron solely for responses to dark bars. Two of the neurons were binocular (Fig. 3a3, c3) and two monocular (Fig. 3b3, d2) for the dark stimulus. Both binocular neurons had their activity peaks for dark bars at a simulated distance of about or close to 26 mm directly in front of the animal (Fig. 3a3, c3) and both monocular neurons were sensitive to the dark bars with their right and thus contralateral eye only (Fig. 3b3, d2; Table 1).

#### Neurons with ramifications in proximal anterior lobe or stalk lobe

We studied five neurons with ramifications in the proximal ALO-V which belonged to three different classes based on their morphology (Fig. 4a1–e1, Table 1). Additionally, we included one cell in our analysis with ramifications in the stalk lobe (SLO) of the LOX (Fig. 4f1). The SLO is located proximal to ALO-V; it is unique to praying mantids and has not been identified in any other insect group (Rosner et al. 2017).

We tested all neuron types with bright flashes of the computer screen (Suppl. Fig. 6). The cells showed clear responses only to the offset of the flash. Thus, we tested all six neurons with dark bars and present results to the onset of the dark bars. With this stimulus, five of the cells were binocular (Fig. 4a2, b2, d2, e2, f2; Table 1,) with mainly excitatory responses and one neuron was monocular (Fig. 4c2; Table 1). This cell was inhibited by left eye stimulation in the left, frontal visual field.

The response fields of the five binocular neurons differed considerably even for neurons of the same morphological type (compare Fig. 4a2 with b2 and c2 with d2). Remarkably, two cells had their main excitatory input from the right and thus contralateral eye where the neurons had no ramifications whatsoever (Fig. 4a2, d2).

### Neurons with ramifications in the medulla

**Fig. 5:**
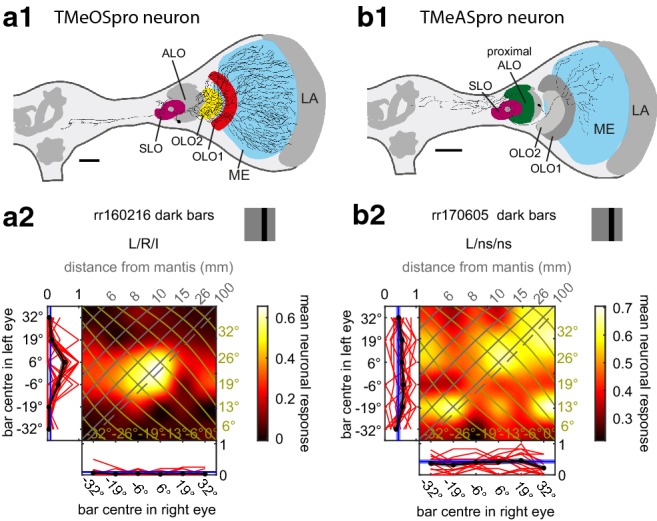
Responses of projection neurons with input in the medulla. **a1**, **b1** Frontal views of reconstructions of neurons with input ramifications in the medulla. As in Fig. 2 the reconstructed neurons are superimposed on the scheme of relevant parts of a praying mantis brain with key neuropils shown. The shape of the brains was adjusted to better capture the location of the neurons within the brain and the neuropils. Optic lobe neuropils with ramifications are coloured. The scale bars are 200 µm. *ALO* anterior lobe, *LA* lamina, *ME* medulla, *OLO1/2* outer lobes 1/2, *SLO* stalk lobe. The ramifications of the TMeASpro neuron in **b1** in the ALO and SLO are strongly beaded as are the ones in the central brain. Thus these are output (presynaptic) ramifications. **a2**, **b2** Monocular and binocular responses of the neurons in **a1** and **b1**. For projection views of reconstructed neurons see Suppl. Fig. 4

We recorded from two neurons with tangential ramifications in the medulla (Fig. 5a1, b1): the second visual neuropil in insects. One of the neurons had additional presumed input ramifications in both outer lobes and in the SLO of the LOX (Fig. 5a1). The neurite projecting into the central brain was only weakly stained but seemed to project to the ventrolateral and inferior neuropils (Table 1). We tested this neuron with dark bars only. It was binocular and tuned to objects directly in front of the animal at a distance of about 10 cm or slightly less (Fig. 5a2). The main excitatory input was from the ipsilateral eye. Monocular right eye stimulation on its own did not cause a change in spiking rate; however, when presented concurrently with left eye stimulation close to the screen centre, the right eye had an inhibitory effect in the periphery (Fig. 5a2).

The second medulla neuron had strongly beaded and thus output ramifications in the dorsal stalk of the optic lobe, in proximal ALO-V and ALO-D regions, in the SLO and in at least a subset of the superior neuropils of the ipsilateral central brain (Fig. 5b1; Suppl. Fig. 4b1–d2). The neuron had complex monocular and binocular response fields without a clear structure (Fig. 5b2). The ANOVA outcome indicated that the cell was monocular for the left (ipsilateral) eye.

## Discussion

We recorded the monocular and binocular response fields of 19 projection neurons of the praying mantis optic lobe. The majority (12/19) of the neurons were demonstrably binocular, i.e. their firing rate could be modulated by input in either eye, from within the frontal area of binocular overlap. Their strongest responses were usually for stimuli in the centre of the frontal visual field (within ± 20° of the midline), and at distances of 2.5–10 cm. Information processing in the lobula complex seems segregated into processing of bright and dark visual input. This is reminiscent of the parallel ON/OFF pathways for moving brightness increments/decrements found in fly optic lobe (Borst et al. 2019).

### Limitations of the study

It needs to be mentioned that we tested only the frontal visual field and our results might be biased with regard to the frequency of occurrence of binocular neurons. On the one hand, it could be that neurons with frontal visual fields are particularly often binocular, and on the other hand, it might be that even neurons that were classed as monocular in our study might actually receive input from the “silent” eye as well in a different, not stimulated part of the visual field. Additionally, visual responsiveness of the mantids’ optic lobe neurons was sometimes abolished by damaging tracheae that supplied the optic lobe and sometimes we even encountered the phenomenon that this abolishment of visual input only affected the optic lobe with the damaged trachea, leaving visual responses from the other eye intact. It was not always possible to see whether a trachea had been damaged and it is thus possible that the percentage of binocular neurons is even higher than reported in this study. Similarly, neurons could appear monocular when tested with bars of one contrast polarity but were revealed as binocular when tested with the opposite polarity (e.g. Fig. 3b2 vs b3). Since not all neurons could be tested with both polarities, this is another possible reason for underestimating the proportion of binocular neurons.

Finally, as noted, we used bar stimuli in order to avoid having to spend time locating the preferred vertical location of a small target. Using long bar stimuli for neurons that might respond preferably to small moving targets, as was recently shown for the TOpro1 neuron presented in Fig. 2a1–3, b1, b2 (Yamawaki 2019), could mask potential responses. However, the response fields generated with our bar stimulus were a very good predictor for sensitivity to small moving targets moving at a particular distance in a recent study (Rosner et al. 2019).

### Segregation of information processing in the lobula complex

Information processing in the praying mantis lobula complex is functionally and spatially segregated. Recent findings (Yamawaki 2019) as well as earlier work by Berger (Berger 1985; Kral and Prete 2004) show that subsets of neurons in the mantis LOX are specialized for small target detection, wide field motion processing and looming detection. Neurons which process information about small dark targets are found in the most distal LOX neuropils, the outer lobes (Rosner et al. 2019; Yamawaki 2019) and in the most proximal LOX neuropil, the SLO (Yamawaki 2019). Widefield motion sensitivity is found in the distal ALO, and the DLO. Looming sensitive neurons have ramifications in a variety of these neuropils. The location of neurons for widefield motion processing and small target detection found by Yamawaki supports earlier suggestions from Rosner et al. (2017) that ALO and DLO could be homologous to the fly lobula plate and the outer lobes the equivalent to the fly lobula. The latter is also strongly supported by the finding of a columnar type of neuron which connects the outer lobes in both LOXs in the praying mantis (COcom-neurons; (Rosner et al. 2019)) and a similar type of neuron which connects both lobulae in flies (LC14 cells, (Hassan et al. 2000; Otsuna and Ito 2006)).

In the current study we also find that neurons which ramify in the distal ALO-V and in the DLO prefer bright stimuli over dark ones. Since all wide-field sensitive neurons found by Yamawaki et al. ramify in the distal ALO-V, DLO or the associated ALO-D, this suggests that wide-field motion sensitivity goes along with a preference for bright contrast in praying mantids. Rosner et al. (2019) suggest that such a preference for bright contrast could, in combination with disparity tuning for far away distances, serve object background segregation because mantids prefer close-by dark prey items in front of a bright background rather than bright targets in front of a distant dark background. While the here recorded neurons with distal ALO-V ramifications seem tuned to distances slightly out of catching range (~ 2.5 cm) when stimulated with bright bars (Fig. 3a2, b2, c2, e2, f2), these distances seem less far away than for the TAcen neuron we reported earlier (Rosner et al. 2019), which also ramifies in the distal ALO-V.

### Functions of binocular neurons

By definition, binocular neurons combine information from both eyes. In principle, there are several reasons why this might be useful to an animal. Perhaps the simplest is where the two eyes contribute information from different regions of space and so binocularity simply enables information to be combined over wider regions of the visual sphere than can be seen by a single eye. Where the two eyes contribute information from overlapping regions of space, binocularity can be useful for improving contrast sensitivity, “seeing round” occluders and providing stereoscopic depth as well as simply redundancy (Changizi and Shimojo 2008; Harris and Wilcox 2009; Blake and Wilson 2011).

### Binocular neurons in invertebrates other than praying mantis

Virtually all previous studies of binocularity in insect neurons have examined only the first of the two situations distinguished in the previous paragraph, i.e. where the two eyes contribute information from non-overlapping directions. Such binocular neurons contribute to the processing of information from optic flow in flies (Hausen 1981; Krapp et al. 2001; Farrow et al. 2006; Huston and Krapp 2008; Wertz et al. 2008). An optic flow sensing neuron with sideways pointing receptive fields can in principle distinguish translation from rotation. If this neuron prefers backwards motion in one eye and forward motion in the other eye then the cell can show its largest response when the animal rotates around its vertical (yaw) axis because it will receive its preferred motion direction in both eyes. In this way it can tell apart rotation and translation because forward translation would cause backwards motion on both eyes which would not stimulate the neuron optimally. Indeed, similar computations have been found to occur in the fly nervous system (e.g. Farrow et al. 2006; Huston and Krapp 2008; Wertz et al. 2008).

Another prominent example of binocular computation enables the so-called FD (figure-detection) cells in blowflies (Egelhaaf 1985) to distinguish movement of a narrow vertical object from rotation of the whole visual surround. A rotation-sensitive binocular neuron of the fly optic lobe inhibits the FD cell when large-field motion occurs in phase with motion detected by the FD cell (Warzecha et al. 1993). Hence, the large-field rotation-sensitive cell enables the FD cell to tell object and background apart.

While binocularity is present among wide field sensitive neurons of the fly optic lobe, most of these cells seem either monocular or rather weakly binocular while binocularity strongly increases at the level of downstream motor- and descending neurons (Krapp et al. 1998, 2001; Huston and Krapp 2008; Wertz et al. 2008, 2012). From the here presented results and from Rosner et al. (2019) it seems that binocularity is more common or more pronounced in optic lobe neurons of the praying mantis than in flies.

Binocular neurons have also been found in other insects, e.g. in butterflies (Schümperli 1975), locusts (Vitzthum et al. 2002; Rosner and Homberg 2013) and dragon flies (Dunbier et al. 2012). Again, in the majority of neurons studied binocularity seems to serve extension of the visual field rather than the computation of depth, which would require that the neurons sample the same region in space with both eyes (Nityananda and Read 2017).

Neurons that might compute depth have been found in a crab (Scarano et al. 2018). The situation in this animal is more complicated than in most insects because the eyes in this invertebrate are not fixed but can move. However, many of the recorded neurons viewed the same region in space when the eyes were fixed in a natural position during the experiments. Thus, these cells could mediate stereopsis in this invertebrate.

### Binocular neurons in vertebrates

In mammals, the neural machinery specific to stereopsis is believed to begin in primary visual cortex (Cumming and DeAngelis 2001; Parker 2007). Although binocular neurons in this area also contribute to other advantages of binocular vision, e.g. improved contrast sensitivity in low light levels (Truchard et al. 2000), they are believed to be specialised to extract stereoscopic depth information, since their properties match those of primate stereopsis in several respects (Cumming and DeAngelis 2001; Parker 2007; Read 2015).

**Fig. 6:**
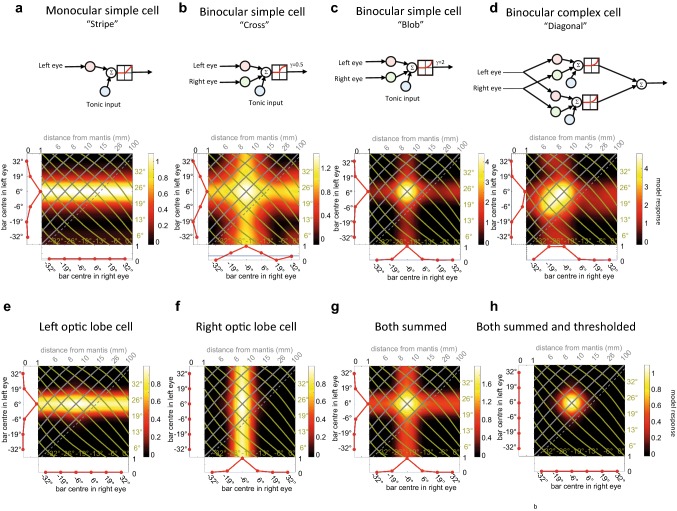
Models of visual neurons, after Ohzawa et al (1990) and Rosner et al. (2019). The model has linear monocular receptive fields in one or both eyes, plus a tonic input, plus a threshold and power-law nonlinearity (see “Materials and methods”). **a**–**d** Processing hierarchy proposed previously for disparity selectivity in mammalian primary visual cortex. **a** Monocular simple cell, receiving input from left eye only. **b**, **c** Binocular simple cell, receiving input from left and right eyes. Receptive fields are identical but shifted so as to give a preference for near objects. In **b** the output nonlinearity is compressive, emphasising the cross structure; in **c** it is expansive, emphasising the blob. **d** Shows a complex cell, consisting of the summed output of two simple cells, **c** tuned to the same disparity but different azimuthal location. **e**–**h** Possible processing hierarchy for disparity selectivity in praying mantis brain. **e**, **f** Binocular neurons in left, right optic lobe. In each case, the ipsilateral receptive field has 10 times the synaptic weight of the contralateral, resulting in strong ipsilateral dominance, and the tonic input is inhibitory. Contralateral input alone is not sufficient to overcome the inhibitory tonic input, so the cell is silent for monocular input in the contralateral eye. However, excitatory input from the contralateral eye can boost the response to excitatory input from the ipsilateral eye. Thus, the stripe is not of uniform height as in **a**, but shows a peak (visible in white) at the preferred location in the contralateral eye. **g** The sum of two such neurons shows a strong preference for stimuli with a particular disparity and azimuthal position, like the mammalian binocular simple cell in **c**. If this is further combined with a threshold, the resulting cell can be made even more selective to disparity. All these models have the same receptive field structure: **a** Gabor function with a standard deviation of 20° and a carrier period of 60°, resulting in an excitatory region surrounded by inhibitory flanking regions. **a**–**d** Receptive fields have equal weights (response to optimal bar = 1 unit) and tonic input is excitatory (*b* =  + 0.1 units); **e**–**h** right-eye receptive field has a weight of 0.1 and tonic input is inhibitory (*b* = − 0.1). Output exponent is *γ* = 1 in **a**, **e**–**h**; *γ* = 0.5 in **b**; *γ* = 2 in **c**–**d**

Stereoscopic neurons in mammalian primary visual cortex have been classified into two major response profiles. Binocular simple cells are roughly equally sensitive to the horizontal location and the binocular disparity, that is, distance of objects, while the further downstream complex cells are more sensitive to depth than to the azimuthal location of an object within their receptive field (Ohzawa et al. 1990). In terms of the binocular response fields like those in our experiments, monocular simple cells are characterised by a horizontal or vertical stripe (Fig. 6a), binocular simple cells by a cross formed by both vertical and horizontal stripes (Fig. 6b), which may be more or less dominated by a blob at their intersection (Fig. 6c), while binocular complex cells are characterised by a diagonal structure indicating greater sensitivity to disparity than to azimuth (Fig. 6d).

### Binocular neurons in praying mantis

The receptive fields of the mantis neurons presented here and in (Rosner et al. 2019) are far larger than those of vertebrate disparity sensitive cells. However, despite this difference in scale, the structure of the binocular response fields is reminiscent of that seen in vertebrate simple cells.

A hallmark of most of the neurons presented here, including those with distal ALO-V ramifications, is strong ocular dominance, manifest as a horizontal or vertical stripe in the binocular response field, with only a rather weak peak of strongest disparity tuning. This is modelled in Fig. 6e, where input from the left eye is given ten times the weight of input from the right. As a result, the response is strong whenever the left eye sees a bar at + 6°, regardless of what is presented in the right eye, visible as a horizontal yellow stripe in the binocular response field. However, there is a small further increase when the right eye also sees a bar at − 6°, visible as a white peak at this location along the stripe. Thus, the cell responds best of all to a binocular object at a distance of 26 mm. In principle, this ocular imbalance could be due to our use of intracellular recordings. Penetrating a cell with a micropipette will unavoidably depolarize it to some extent, at least temporarily, and potentially increase its spiking rate by unmasking sub-threshold input. However, the neuron presented in Fig. 5a and the several neurons shown by Rosner et al. (2019) with stronger disparity tuning suggest that this weaker disparity tuning is a property of specific cell types, rather than a general feature due to our recording technique.

We speculate that there are not only parallel pathways for disparity calculation and thus stereoscopic vision but that this process could consist of multiple steps with several nonlinearities strengthening disparity tuning. If neurons from both sides of the brain provide input to the same descending or motor neurons, then monocular preference will be annihilated (see model example in Fig. 6g) and applying a spiking threshold nonlinearity would further fine tune disparity sensitivity (see Fig. 6h). This would be analogous to the circuitry believed to exist in primate visual cortex, where disparity selectivity is first established in simple cells and then is refined and strengthened when multiple simple cells converge onto a complex cell (Fig. 6d).

Here again a parallel exists with optic flow processing in the fly. Binocularity is increased in motor neurons and descending neurons and serves for strengthening sensitivity to rotational flow fields (Huston and Krapp 2008; Wertz et al. 2008). Thus, the same neuronal connections and computations which fine-tune binocular optic flow processing in flies might boost disparity tuning and stereoscopic vision in the praying mantis.

## Electronic supplementary material

Below is the link to the electronic supplementary material.

Supplementary Fig. 1 Sample images of stained neurons used for neuron reconstructions in Fig. 2. a1, Scheme of excerpt of left mantis brain hemisphere as shown in Fig. 2 a1. a2, Projection view of multiple confocal images with stained TOpro1 neuron. a3, Projection view with superimposed reconstruction of TOpro1 neuron. b1-e3, Schemes, projection views and projection views with superimposed reconstructions of all other neurons shown in Fig. 2. The ID of each neuron is provided as header, respectively. All scale bars are 200µm (JPG 5385 kb)

Supplementary Fig. 2 Sample images of stained neurons used for neuron reconstructions in Fig.3. a1, Scheme of excerpt of left mantis brain hemisphere as shown in Fig. 3 a1. a2, Projection view of multiple confocal images with stained TAprodistX neuron. a3, Projection view with superimposed reconstruction of TAprodistX neuron. b1-f3, Schemes, projection views and projection views with superimposed reconstructions of all other neurons shown in Fig. 3. The ID of each neuron is provided as header, respectively. All scale bars are 200µm (JPG 6607 kb)

Supplementary Fig. 3 Sample images of stained neurons used for neuron reconstructions in Fig.4. a1, Scheme of excerpt of left mantis brain hemisphere as shown in Fig. 4 a1. a2, Projection view of multiple confocal images with stained TAproproxt1 neuron. a3, Projection view with superimposed reconstruction of TAproproxt1 neuron. b1-f3, Schemes, projection views and projection views with superimposed reconstructions of all other neurons shown in Fig. 4. The ID of each neuron is provided as header, respectively. All scale bars are 200µm (JPG 5216 kb)

Supplementary Fig. 4 Images of stained neurons used for neuron reconstructions in Fig.5. a1, Scheme of excerpt of left mantis brain hemisphere as shown in Fig. 5 a1. a2, Projection view of multiple confocal images with stained TMeOSpro neuron. a3, Projection view with superimposed reconstruction of TMeOSpro neuron. b1-b3, Scheme, projection view and projection view with superimposed reconstruction of TMeASpro neuron also shown in Fig. 5b1. c1-d2 Documentation of beaded neurites in optic lobe and central brain of TMeASpro neuron. Red arrows in c2, c3, c4 and circle in d2 indicate beaded and thus presumed presynaptic regions. Refer to neuron reconstruction in b1,c1 and d1 for navigating the images. Scale bars are 200µm (JPG 6044 kb)

Supplementary Fig. 5 Neurons with ramifications in distal ALO layers respond to bright contrast and/or contrast increments. a,c,d,f Responses of the neurons shown in Fig. 3a1,c1,d1,f1 to whole screen flashes. Top panels show membrane potential and bottom panels spiking rate estimated with Gaussian filter (SD 50ms). Bright screen time periods are highlighted cyan and time periods with dark screen are highlighted grey. The neurons respond strongest when the contrast changes from dark to bright. b,e Recording trace excerpts of responses to bright (left panels) and dark (right panels) bar flashes for two neurons for which we did not show whole screen flashes (neurons from Fig. 3 b1 and e1). Bight bar flashes are indicated cyan and dark bar flashes in grey. The remainder of the screen and in pauses the entire screen were at intermediate brightness (grey). The bar locations were exactly the same in left and right panels; only the contrast was different. The neuron in b responds with spikes only to the onset of bright bar flashes (left panel) and to the offset of dark bar flashes (right panel). The neuron in e responds with the highest spiking rates to bright bar flashes (compare left and right panels). The ID of each neuron and the stimulus is provided asw header, respectively (PDF 2613 kb)

Supplementary Fig. 6 Neurons with ramifications in proximal ALO layers respond to dark contrast and/or contrast decrements. a,b,c,e Responses of the neurons shown in Fig. 4b1,c1,d1,f1 to whole screen flashes. Top panels show membrane potential and bottom panels spiking rate estimated with Gaussian filter (SD 50ms). Bright screen time periods are highlighted cyan and time periods with dark screen are highlighted grey. The neurons respond strongest when the contrast changes from bright to dark. d Cell responses to an entire presentation of all 48 bright bar conditions (entire time period with flashes and pauses cyan) and 48 dark bar flashes (entire time period with flashes and pauses grey) for a neuron for which we did not show whole screen flashes (neuron from Fig. 4 e1. The neuron showed pronounced spiking rate changes only during dark bar presentation. The ID of each neuron and the stimulus is provided as header, respectively (PDF 1926 kb)

## Abbreviations

ALO: Anterior lobe of the lobula complex (ALO-V,D = ventral, dorsal subunits)
DLO: Dorsal lobe of the lobula complex
INP: Inferior neuropils
LA: Lamina
LOX: Lobula complex
ME: Medulla
OL: Optic lobe
OLO: Outer lobe of the lobula complex (OLO1, OLO2 = 1st, 2nd neuropil from distally)
SLO: Stalk lobe of the lobula complex
VLNP: Ventrolateral neuropils
VMNP: Ventromedial neuropils
