# Supplementary figures and images for: Binocular responsiveness of projection neurons of the praying mantis optic lobe in the frontal visual field
Source: J Comp Physiol A Neuroethol Sens Neural Behav Physiol. 2020 Feb 22;206(2):165–81. doi: 10.1007/s00359-020-01405-x (PMC7069917; doi:10.1007/s00359-020-01405-x)

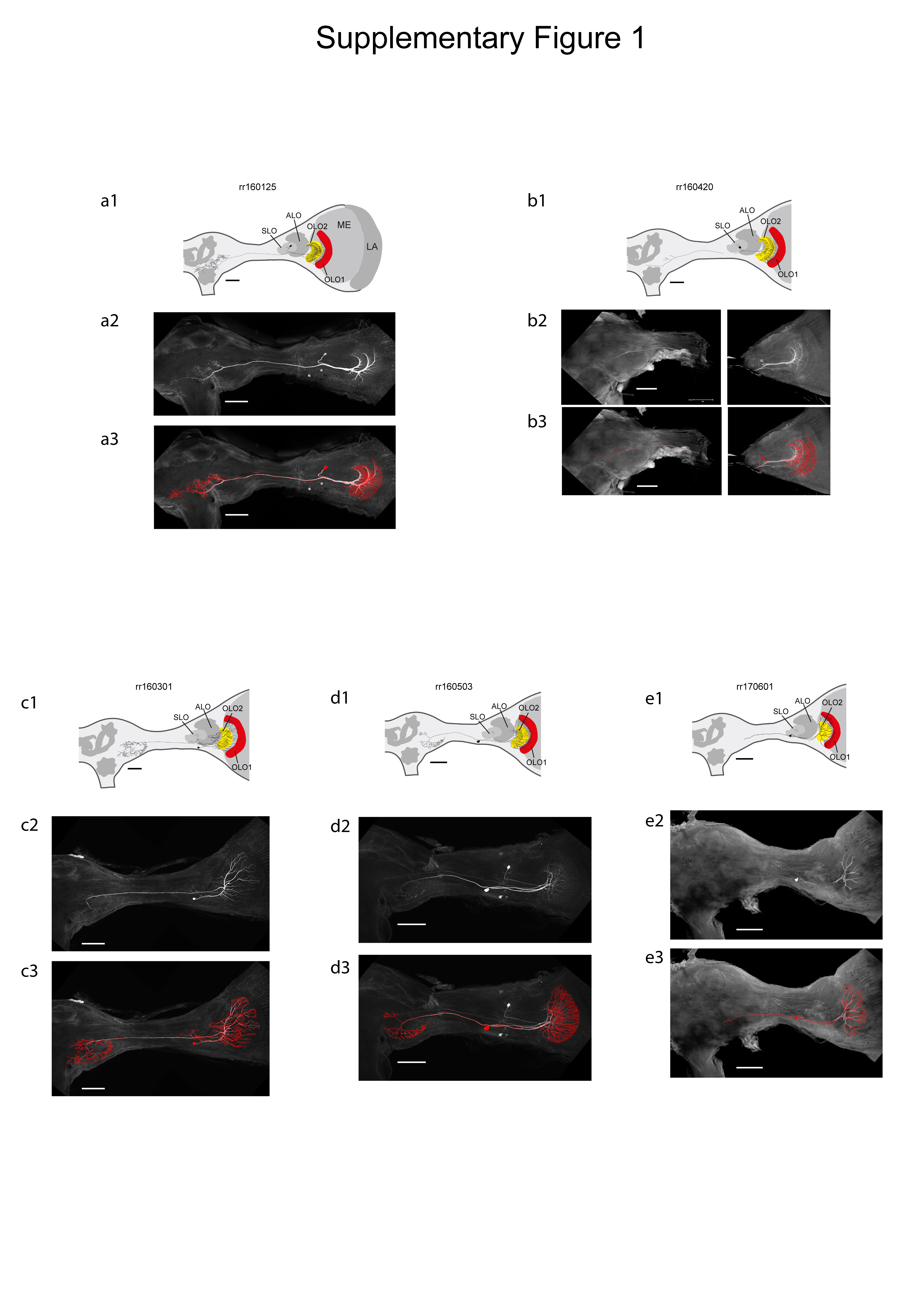

Supplement: Supplementary file 1 — Supplementary Fig. 1 Sample images of stained neurons used for neuron reconstructions in Fig. 2. a1, Scheme of excerpt of left mantis brain hemisphere as shown in Fig. 2 a1. a2, Projection view of multiple confocal images with stained TOpro1 neuron. a3, Projection view with superimposed reconstruction of TOpro1 neuron. b1-e3, Schemes, projection views and projection views with superimposed reconstructions of all other neurons shown in Fig. 2. The ID of each neuron is provided as header, respectively. All scale bars are 200µm (JPG 5385 kb) [file 359_2020_1405_MOESM1_ESM.jpg]

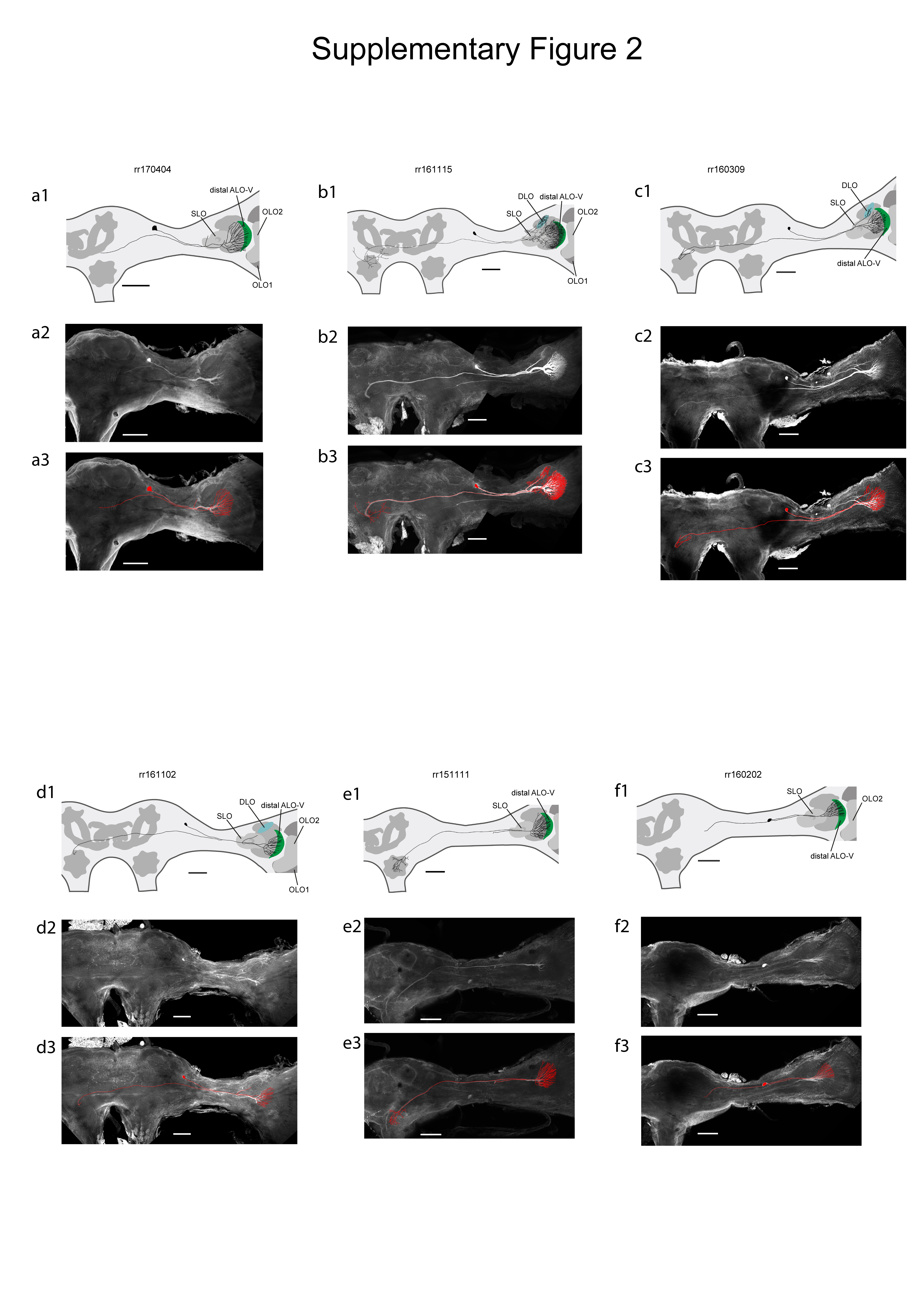

Supplement: Supplementary file 2 — Supplementary Fig. 2 Sample images of stained neurons used for neuron reconstructions in Fig.3. a1, Scheme of excerpt of left mantis brain hemisphere as shown in Fig. 3 a1. a2, Projection view of multiple confocal images with stained TAprodistX neuron. a3, Projection view with superimposed reconstruction of TAprodistX neuron. b1-f3, Schemes, projection views and projection views with superimposed reconstructions of all other neurons shown in Fig. 3. The ID of each neuron is provided as header, respectively. All scale bars are 200µm (JPG 6607 kb) [file 359_2020_1405_MOESM2_ESM.jpg]

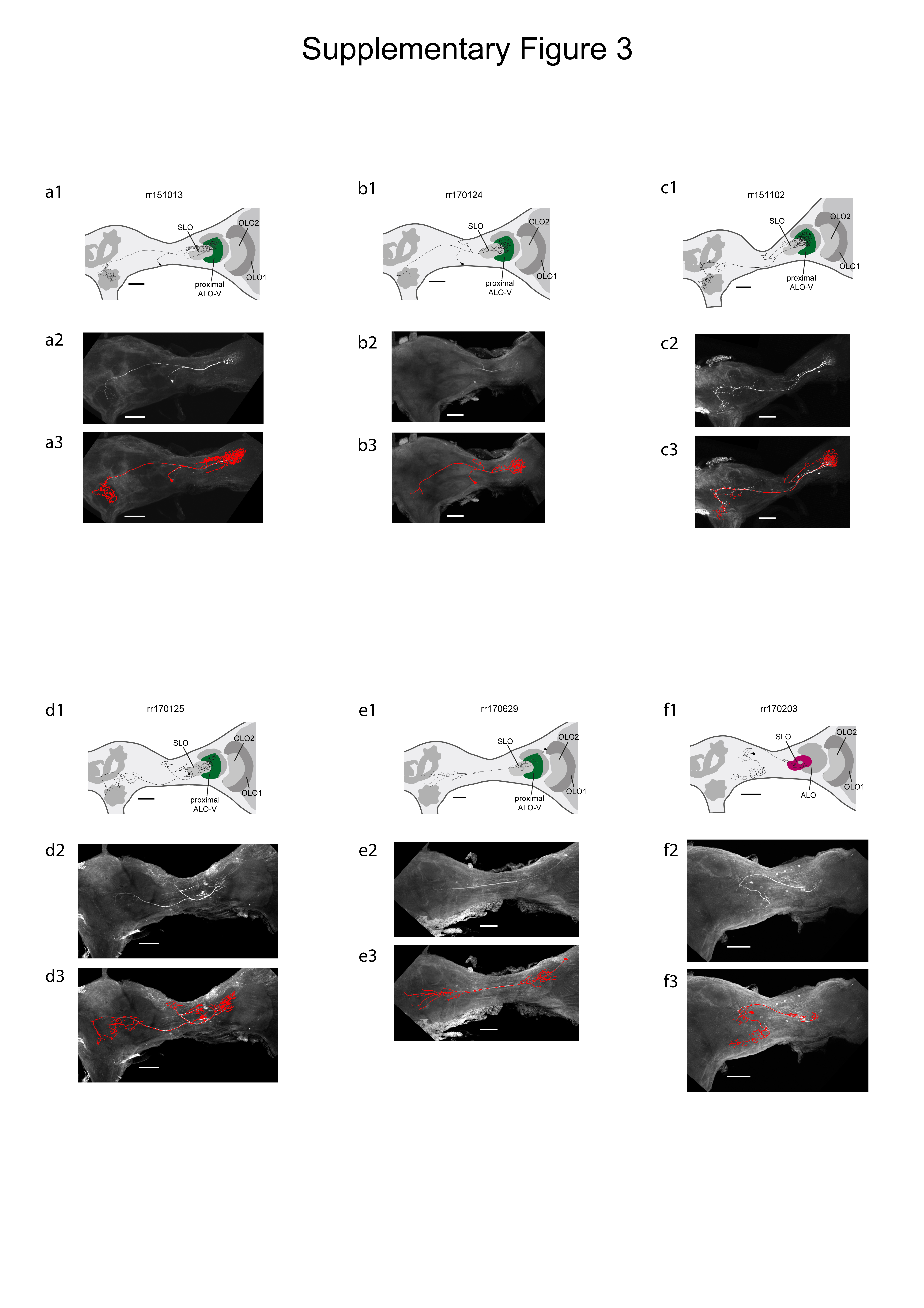

Supplement: Supplementary file 3 — Supplementary Fig. 3 Sample images of stained neurons used for neuron reconstructions in Fig.4. a1, Scheme of excerpt of left mantis brain hemisphere as shown in Fig. 4 a1. a2, Projection view of multiple confocal images with stained TAproproxt1 neuron. a3, Projection view with superimposed reconstruction of TAproproxt1 neuron. b1-f3, Schemes, projection views and projection views with superimposed reconstructions of all other neurons shown in Fig. 4. The ID of each neuron is provided as header, respectively. All scale bars are 200µm (JPG 5216 kb) [file 359_2020_1405_MOESM3_ESM.jpg]

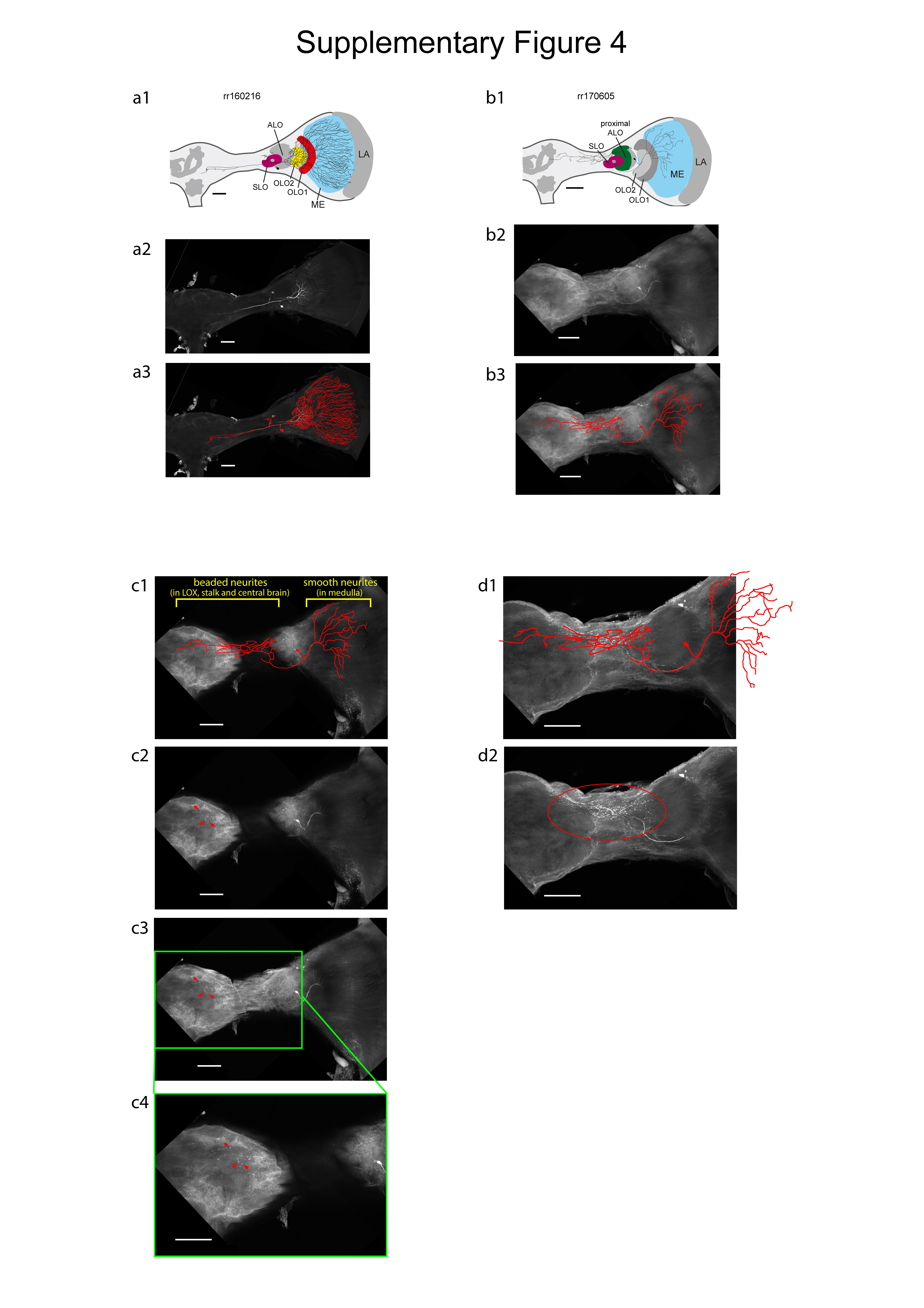

Supplement: Supplementary file 4 — Supplementary Fig. 4 Images of stained neurons used for neuron reconstructions in Fig.5. a1, Scheme of excerpt of left mantis brain hemisphere as shown in Fig. 5 a1. a2, Projection view of multiple confocal images with stained TMeOSpro neuron. a3, Projection view with superimposed reconstruction of TMeOSpro neuron. b1-b3, Scheme, projection view and projection view with superimposed reconstruction of TMeASpro neuron also shown in Fig. 5b1. c1-d2 Documentation of beaded neurites in optic lobe and central brain of TMeASpro neuron. Red arrows in c2, c3, c4 and circle in d2 indicate beaded and thus presumed presynaptic regions. Refer to neuron reconstruction in b1,c1 and d1 for navigating the images. Scale bars are 200µm (JPG 6044 kb) [file 359_2020_1405_MOESM4_ESM.jpg]

# Supplementary Figure 5

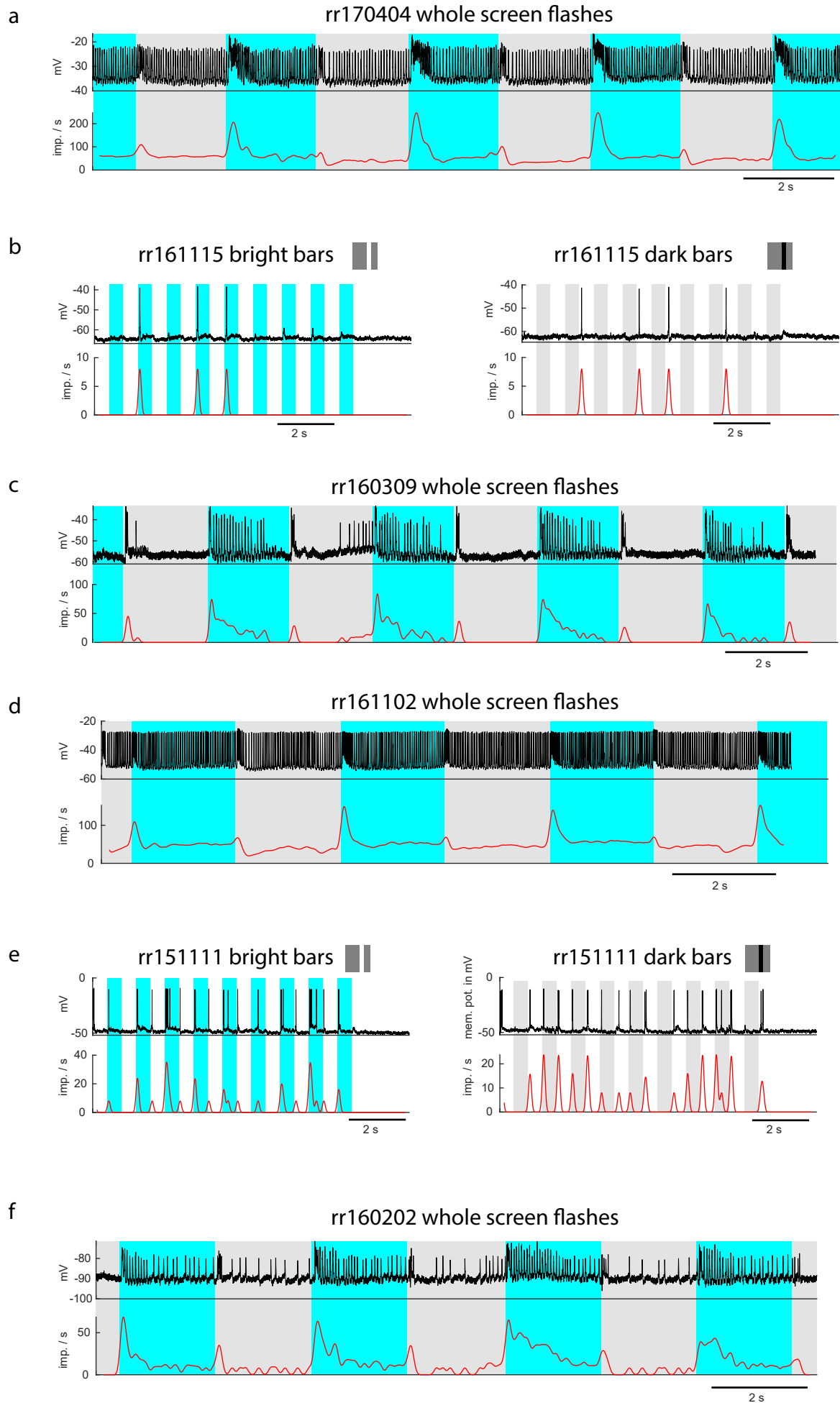

Supplement: Supplementary file 5 — Supplementary Fig. 5 Neurons with ramifications in distal ALO layers respond to bright contrast and/or contrast increments. a,c,d,f Responses of the neurons shown in Fig. 3a1,c1,d1,f1 to whole screen flashes. Top panels show membrane potential and bottom panels spiking rate estimated with Gaussian filter (SD 50ms). Bright screen time periods are highlighted cyan and time periods with dark screen are highlighted grey. The neurons respond strongest when the contrast changes from dark to bright. b,e Recording trace excerpts of responses to bright (left panels) and dark (right panels) bar flashes for two neurons for which we did not show whole screen flashes (neurons from Fig. 3 b1 and e1). Bight bar flashes are indicated cyan and dark bar flashes in grey. The remainder of the screen and in pauses the entire screen were at intermediate brightness (grey). The bar locations were exactly the same in left and right panels; only the contrast was different. The neuron in b responds with spikes only to the onset of bright bar flashes (left panel) and to the offset of dark bar flashes (right panel). The neuron in e responds with the highest spiking rates to bright bar flashes (compare left and right panels). The ID of each neuron and the stimulus is provided asw header, respectively (PDF 2613 kb) [file 359_2020_1405_MOESM5_ESM.pdf]

# Supplementary Figure 6

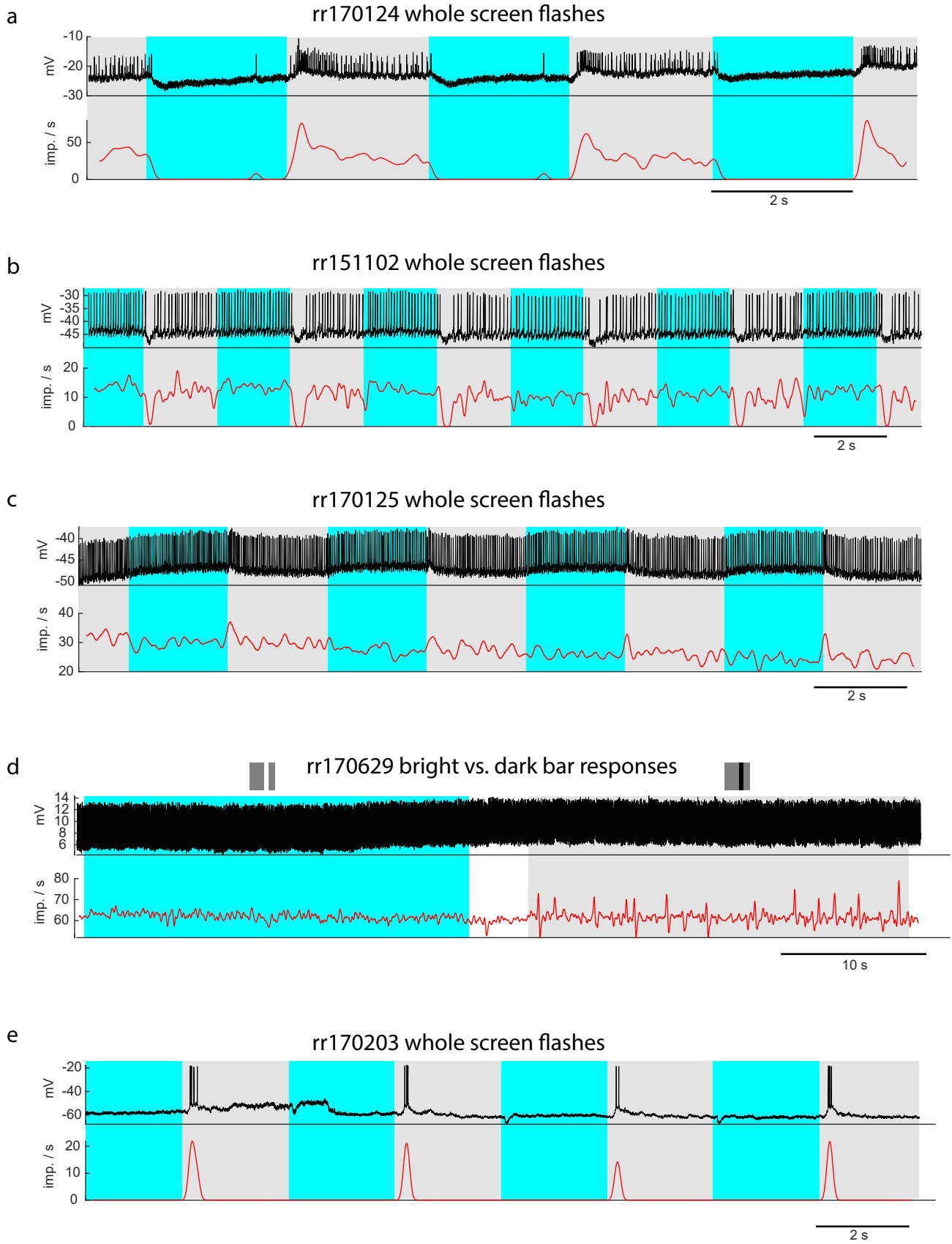

Supplement: Supplementary file 6 — Supplementary Fig. 6 Neurons with ramifications in proximal ALO layers respond to dark contrast and/or contrast decrements. a,b,c,e Responses of the neurons shown in Fig. 4b1,c1,d1,f1 to whole screen flashes. Top panels show membrane potential and bottom panels spiking rate estimated with Gaussian filter (SD 50ms). Bright screen time periods are highlighted cyan and time periods with dark screen are highlighted grey. The neurons respond strongest when the contrast changes from bright to dark. d Cell responses to an entire presentation of all 48 bright bar conditions (entire time period with flashes and pauses cyan) and 48 dark bar flashes (entire time period with flashes and pauses grey) for a neuron for which we did not show whole screen flashes (neuron from Fig. 4 e1. The neuron showed pronounced spiking rate changes only during dark bar presentation. The ID of each neuron and the stimulus is provided as header, respectively (PDF 1926 kb) [file 359_2020_1405_MOESM6_ESM.pdf]
